# Endometriosis Longitudinal Fertility Study (ELFS): a prospective longitudinal cohort study of the effect of conservative and surgical management for moderate to severe endometriosis on future fertility – study protocol

**DOI:** 10.1136/bmjopen-2025-114124

**Published:** 2026-08-20

**Authors:** Samantha Mooney, Vanessa Ross, Uri P Dior, Charlotte Reddington, Lauren Hicks, Claudia Cheng, Michal Amir, Keryn Harlow, Sarah Holdsworth-Carson, Kate Stern, Martin Healey, Peter Rogers, END-IT Endometriosis Research Group

**Affiliations:** 1 Gynaecology and Endosurgery Departments, Mercy Hospital for Women, Heidelberg, Victoria, Australia; 2 Department of Obstetrics, Gynaecology and Newborn Health, The University of Melbourne, Melbourne, Victoria, Australia; 3 Julia Argyrou Endometriosis Centre, Epworth HealthCare, Richmond, Victoria, Australia; 4 Heidelberg Clinic, Genea Fertility, Heidelberg, Victoria, Australia; 5 Melbourne IVF, Melbourne, Victoria, Australia; 6 Reproductive Services and Gynaecology 2 Units, The Royal Women’s Hospital, Parkville, Victoria, Australia; 7 Gynaecology, Hadassah Medical Center - Hadassah Hospital, Jerusalem, Israel; 8 Newlife Fertility, Box Hill, Victoria, Australia; 9 Gynaecology, Te Whatu Ora Health New Zealand, Christchurch, New Zealand; 10 Fertility Associates, Christchurch, New Zealand; 11 The Royal Women’s Hospital, Parkville, Victoria, Australia; 12 Reproductive Services Unit, Royal Women’s Hospital, Parkville, Victoria, Australia

**Keywords:** Minimally invasive surgery, Reproductive medicine, Subfertility, Adult surgery

## Abstract

**Introduction:**

Endometriosis is associated with reduced pregnancy and live birth rates in patients attempting both spontaneous and assisted conception. At present, it is unclear whether prepregnancy surgery for moderate or severe endometriosis, either in the setting of established infertility or even before a woman has begun trying to conceive, confers any benefit in terms of (a) spontaneous conception, (b) conception with assisted reproductive technology (ART), (c) live birth or (d) other obstetric outcomes.

**Methods and analysis:**

The Endometriosis Longitudinal Fertility Study (ELFS) is a multisite prospective longitudinal cohort study, recruiting patients <38 years of age with evidence of moderate or severe endometriosis (revised American Society of Reproductive Medicine grades 3 or 4) who desire fertility immediately or in the near future. ELFS aims to longitudinally assess clinical pregnancy and live birth rates for women with moderate or severe endometriosis who choose to have no treatment, fertility treatment and/or surgery. The target sample size was determined by a simulation-based power calculation. The study will comprise 700 participants: 400 attempting natural conception and 300 attempting to conceive with ART. Each of these cohorts will be divided evenly between the conservative and surgical management groups. Following consent and baseline questionnaire, participants instal the purpose-built ELFS App to their mobile phone. The participant then receives cyclical surveys at intervals based on their menstrual cycle, with timing dependent on learnt logic from prior responses. Participants conclude participation following a live birth beyond 20-week gestation. The primary research outcome is to compare the ‘per cycle’ clinical pregnancy rate and live birth rate where endometriosis is managed conservatively or surgically in those conceiving naturally or with assisted reproductive techniques. Secondary outcomes include exploring menstrual cycle characteristics and examining the obstetric outcomes of women with moderate and severe endometriosis who successfully conceive. The outcomes of this research aim to contribute to improving endometriosis management for women desiring pregnancy by establishing if there is a fertility benefit of prepregnancy surgery in patients with moderate or severe endometriosis.

**Ethics and dissemination:**

The Austin Health Ethics Committee approved the ELFS protocol (HREC/65683/Austin-2020). On study completion, results of the trial will be submitted for publication in a peer-reviewed journal. Interim experiences with the ELFS App as well as relevant secondary outcomes will be presented at international conferences with additional goals for publication.

**Trial registration number:**

ACTRN12621000431820.

**STRENGTHS AND LIMITATIONS OF THIS STUDY**

- The current evidence examining the role of prepregnancy surgery for moderate and severe endometriosis is predominantly retrospective. A main strength of this study is its prospective pragmatic cohort design.
- The study will use a novel tool (the Endometriosis Longitudinal Fertility Study App) to attempt to improve longitudinal follow-up rates and participant engagement, recognising that endometriosis is a chronic condition and many participants will have prolonged fertility journeys.
- The study will run internationally and use translation technologies to enable recruitment of language-diverse populations.
- The primary outcome requires a large study design. Recruitment rates may be impacted by strict inclusion criteria required to ensure accuracy in the diagnosis of moderate-severe endometriosis.
- Repeated collection of sensitive data (eg, sexual history, fertility, pregnancy outcomes) may have a negative impact on participant experience and engagement.

## Introduction

 Epidemiological studies report a strong association between infertility and subsequent endometriosis diagnosis,^1 2^ and 30%–50% of women with endometriosis experience difficulties conceiving.^3 4^ This association may reflect biological effects of endometriosis on fertility, increased likelihood of diagnostic evaluation among women with infertility or both. Surgically treating milder endometriosis (revised American Society of Reproductive Medicine (rASRM) stages 1 and 2)^5^ has been demonstrated to improve spontaneous clinical pregnancy rates (CPRs) in prospective randomised controlled trials^6^ and is the topic of a recent Cochrane review.^7^ However, for women planning or desiring fertility, the role of surgery for moderate and severe endometriosis (including deep endometriosis and ovarian endometriosis) in improving fertility and pregnancy outcomes is not fully understood.^7–16^

A literature review^17^ summarising the fertility outcomes before and after surgery for deep endometriosis concluded that in patients without bowel involvement, postoperative overall pregnancy rates were 68.3% (95% CI 64.9% to 71.7%) but no comparison with presurgical pregnancy rates was possible. In the same review, patients with bowel endometriosis may have experienced improved pregnancy rates after surgery, but numbers were small and all conceived with assisted reproductive technology (ART). Daniilidis *et al*^15^ published a systematic review examining the impact of prepregnancy surgery on subsequent fertility. The authors reported inconsistent outcomes and concluded that more studies are needed to ascertain whether surgical management should be used as first line or only in cases where ART has failed. Similarly, Liang *et al*^10^ published their review comparing pregnancy rates between first line surgery for deep endometriosis and first line ART. The authors reported comparable outcomes between the groups and concluded that further studies were needed, particularly with regard to surgical management of endometrioma. Thus, at present, the European Society of Human Reproductive Endocrinology guidance advises that operative laparoscopy may be a treatment choice for symptomatic patients who want to become pregnant, but no compelling evidence exists to support surgical treatment of deep endometriosis to improve spontaneous or ART pregnancy rates.^18^

Laparoscopic surgery to remove endometriosis exposes women to surgical risks. Surgery could be justified if there is a potential benefit, either in symptom reduction or with improved fertility. Current evidence suggests that patients with deep endometriosis may have improvements in pain symptoms following surgery, particularly after colorectal surgery.^19^ In the group of patients who do not experience significant pain and would be considering surgery to optimise fertility alone, further evidence is needed before the risks of surgery can be justified. Moreover, the issue of future fertility is a vexed question for many women with endometriosis, and the known association with infertility raises questions about whether pre-emptive surgical treatment of endometriosis will provide future benefit with improved pregnancy rates, particularly with regard to risk of adhesions, prevention of disease progression and reductions in ovarian reserve.^20 21^

In recent years, there has also been an increase in research focused on the association between endometriosis and pregnancy outcome.^22–24^ It is possible that the presence of endometriosis, or history of a prior diagnosis of endometriosis, may be associated with poor pregnancy outcomes^25–27^ including placenta praevia, preterm birth, premature prelabour rupture of membranes, obstetric haemorrhage, gestational hypertensive disorders, fetal growth restriction and perinatal death.^28^ It is unknown whether the obstetric outcome risk is ameliorated by prepregnancy surgery for endometriosis or if there is a surgically untreatable factor which underpins the likely increased risk of adverse obstetric outcomes.^9^

Ultimately, our proposed study aims to compare fertility outcomes among four groups of participants with evidence of moderate-severe endometriosis (EMSE):

- Those pursuing natural conception without prior surgery.
- Those pursuing ART without prior surgery.
- Those undergoing surgery followed by attempts at natural conception.
- Those undergoing surgery followed by ART.

Cross-comparisons are planned across all four groups.

This study will explore the impact of surgical treatment of moderate to severe endometriosis on pregnancy and live birth rates (LBRs) from both natural conception and in vitro fertilisation (IVF). Obstetric and perinatal outcomes will be assessed as secondary outcomes. In addition, it will seek to clarify whether pre-emptive surgical treatment of endometriosis has an impact on future fertility.

## Methods and analysis

The Endometriosis Longitudinal Fertility Study (ELFS) is a prospective longitudinal cohort study.

### Study objectives

ELFS aims to measure and compare monthly clinical pregnancy and LBRs prospectively and longitudinally in women with EMSE who are undertaking surgery or conservative management prior to conception. Secondary objectives include collection of menstrual cycle data longitudinally in women with moderate or severe endometriosis.

Primary and secondary outcomes have been prespecified with explicit definitions.

#### Primary outcome

LBR (live birth/number of attempted cycles)—‘live birth’ will be defined as birth of a live infant >20 weeks gestation.

For women with EMSE who are attempting natural conception, the ‘per cycle’ LBR will be compared between conservatively or surgically managed groups.

For women with EMSE and infertility who are undergoing IVF, the ‘per cycle’ LBR will be compared between conservatively or surgically managed groups.

#### Secondary outcomes

1. CPR (clinical pregnancy/number of attempted cycles)—for the purposes of this study, ‘clinical pregnancy’ will be defined as a pelvic ultrasound demonstrating a gestational sac (intrauterine or extrauterine).
  For women with EMSE who have been trying to conceive for less than 12 months or plan to in the future, the ‘per cycle’ CPR will be compared between groups where EMSE is managed conservatively or surgically excised.
  For women with EMSE and infertility who are undergoing IVF the ‘per cycle’ CPR will be compared between groups where EMSE is managed conservatively or surgically excised.
  For women with EMSE and infertility who are attempting natural conception, the ‘per cycle’ CPR will be compared between groups where EMSE is managed conservatively or surgically excised.
2. Menstrual cycle characteristics including: cycle length, length of menstrual period, heaviness of flow, associated pain symptoms.
3. Rates of adverse pregnancy outcomes of women with EMSE who successfully conceive. This is a composite outcome of adverse perinatal outcome including—premature birth, hypertensive disorders of pregnancy, caesarean delivery, fetal growth restriction and severe maternal mortality.

### Recruitment

Patients of public hospitals in Australia (Royal Women’s Hospital, Mercy Hospital for Women, Joan Kirner Sunshine Hospital Western Health and Royal Prince Alfred Hospital) and at Hassadah Medical Centre (Jerusalem, Israel), along with patients receiving care at private institutions (Ramsay Health, Epworth HealthCare, Melbourne IVF, NewLife IVF, Fertility (Sydney CBD and Melbourne CBD), Genea Fertility and private consulting rooms of affiliated gynaecologists) are screened for eligibility. Accredited study team members (gynaecologists, gynaecology trainees and research nurses/assistants) will screen outpatient clinic lists, surgery waiting lists and medical records, and identify patients during consultations, and preadmission clinics. Eligible participants may be approached in person, by phone or email; treating clinicians may record verbal expressions of interest and refer these to research nurses/assistants, who will confirm eligibility, manage consent and oversee recruitment. Advertising material describing the project is available in waiting rooms of participating hospital clinics and private gynaecologists’ rooms. A website (https://www.endometriosis.org.au) enables patients to access information about the study online and to enquire about eligibility.

Potential participants are provided with written and verbal information about the study. Those wishing to participate will provide consent either in written form or via an online consent managed by REDCap (Research Electronic Data Capture; Vanderbilt University, Nashville, Tennessee, USA).^29 30^ Individual consent is obtained from each participant prior to any information being gathered. Consent from male partners is requested, recognising the potential for comorbid male-factor infertility. If indicated, semen analysis reports will be sought as additional data.

### Sample selection: inclusion and exclusion criteria

#### Inclusion criteria

- ≥18 years and <38 years of age at time of recruitment. These age restrictions were made due to (a) jurisdictional issues with the recruitment of minors and (b) local and international practices where ART is more likely to be recommended to women of advanced age seeking conception, irrespective of the cause of the infertility.
- Desire for fertility (current or future) including women with infertility
- EMSE—defined as
  - Ultrasonically (expert tertiary level sonologist) or MRI diagnosed endometriosis includes current presence of:
    - An ovarian cyst with ground-glass appearance that is the same size or larger on repeat imaging 4 weeks or more apart.
    - Deep infiltrating endometriosis (deep to peritoneum) or other hallmarks (such as obliterated Pouch of Douglas) as diagnosed on ultrasound or MRI by a practitioner with the required skills.
  - Presence of untreated moderate or severe endometriosis (rASRM stages 3 or 4) on a previous diagnostic laparoscopy/laparotomy.
- Own and use a mobile phone.

#### Exclusion criteria

- Unable to understand and fully comprehend consent information.
- Do not wish to have future fertility.
- Findings suspicious of malignancy on expert imaging.
- Current pregnancy.

At the time of protocol development, no validated non-surgical preoperative grading system existed; the sonographic #ENZIAN classification was only published and validated in 2021. Because diagnostic laparoscopy without treatment is uncommon in clinical settings and requiring surgical confirmation would have severely limited recruitment, a pragmatic decision was made to use expert pelvic ultrasound and/or MRI to identify participants with imaging evidence of moderate–severe disease. We acknowledge that applying rASRM^5^ criteria to imaging is not validated and is a limitation; however, we minimised misclassification through strict imaging-provider protocols and predefined imaging criteria for moderate–severe endometriosis, and we will analyse the surgical subgroup to confirm concordance with rASRM stages 3–4, which will support the validity of our imaging-based cohort.^31^

#### Study groups

Participants are allocated to one of two management groups prior to conception: a preconception surgery group (n=350) or a planned non-surgical (conservative) management group (n=350). Within each management group, participants will be subdivided by conception method into a natural conception subgroup (n=200 in each of the surgical and conservative management groups) and an ART (ART/IVF) subgroup (n=150 per preconception management group). Participants may contribute multiple attempted conception cycles, and to both natural and ART cycles.

### Interventions

Participants will complete an initial patient questionnaire focusing on demographic and fertility data. This baseline data are collected and managed using REDCap electronic data capture tools hosted at The University of Melbourne.^29 30^ REDCap is a secure, web-based software platform designed to support data capture for research studies, providing (1) an intuitive interface for validated data capture; (2) audit trails for tracking data manipulation and export procedures; (3) automated export procedures for seamless data downloads to common statistical packages and (4) procedures for data integration and interoperability with external sources. Additional clinical information will be collated by the research assistant team, ensuring systematic documentation of endometriosis imaging and/or prior surgical findings, prior fertility investigations (if relevant) and choice of management pathway.

A mobile phone application (known as an ‘App’) has been developed as a data collection tool specific to this study—the ELFS App. The original App was free for participants to download from the Apple App Store and Google Play (December 2021—November 2024). A newly developed version of the ELFS App is designed as a Progressive Web App which is installed on the participants’ mobile device as an application but is deployed by, and retains the functionality of, a website. Transition to the new App occurred over a fortnight period in November 2024. Participant data and survey responses from the first system were exported, then custom scripts used to transform and validate the information before importing it into the new App platform.

Participants are assisted by the study team to set-up the App, which is then used for cyclical survey data collection. Participants complete a monthly/cyclical questionnaire via the mobile App (or they may choose to complete via the online website) to record information about: (a) pregnancy status; (b) whether or not conception was attempted; (c) method of attempt and (d) outcome of pregnancy (if relevant). The participants also complete a menstrual calendar (on the original ELFS App) or a menstrual symptom questionnaire (on the new ELFS App 2.0) each cycle. If the participant does not complete the questionnaire, an SMS or email reminder will be sent to them after 3 days of inactivity. If the questionnaire is still not completed, a second reminder will be sent after a further 3 days. If there is no response after these two reminders, the cycle will be ‘skipped’. If a participant does not complete a survey for three consecutive months, they will be contacted by the research team to confirm their intention to continue within the study.

A study-specific, cycle-based questionnaire was designed to capture (1) detailed menstrual-cycle characteristics and related events (eg, cycle duration and regularity, intercourse timing, pregnancy attempts and contraceptive use), (2) relationship and demographic factors relevant to conception and (3) reproductive outcomes including conception and live birth. Because existing validated instruments do not, to our knowledge, combine these two aims in a brief, per cycle format, we created an adaptive (logic-driven) instrument that presents only questions relevant to each participant in a given cycle. The instrument is intentionally concise because surveys are administered frequently (approximately monthly) and the primary outcomes of the study are conception and obstetric endpoints rather than comprehensive symptom profiling.

Menstrual-cycle symptoms are measured using a simple, 4-point Verbal Rating Scale (VRS) for pain intensity (no pain/mild pain/moderate pain/severe pain) accompanied by pictograms to improve respondent interpretation and reduce response burden. VRSs are widely used and have demonstrated acceptable validity and reliability for clinical pain intensity assessment compared with other pain measures (eg, Visual Analogue and Numeric Rating Scales) and are well suited to brief, repeated measurement in field studies.^32^ A brief 4-point VRS was included rather than longer symptom inventories because more comprehensive menstrual symptom instruments (eg, the Menstrual Distress Questionnaire and other multi-item menstrual symptom scales) are lengthy and were not feasible for monthly administration in a fertility cohort in which the principal outcomes are reproductive.^33^ The questionnaire items and the VRS pain measure were selected to maximise face validity, minimise respondent burden, and enable longitudinal linkage of symptom reports to timing of intercourse, ovulation windows, pregnancy test results and pregnancy outcomes.

The accuracy of participant-entered clinical data is safeguarded by the structured nature of the study-specific questionnaires and real-time front-end checks to prevent entry errors, automated rules that flag implausible or inconsistent responses for in-app confirmation and staff follow-up for clarification. Where participants consent, medical records will be reviewed to verify key outcomes such as surgical reports. For those undergoing surgery during the study period, surgical reports will be collected and assessed by one of two investigators (SM and VR); any disagreements will be clarified by investigator MH. The primary outcome, LBR, is assessed by patient reported data (via the ELFS App) and cross-validated against obstetric outcome data retrieved from the hospital in which the birth occurs. Similarly, CPR is assessed by patient report, and validated by review of first trimester sonographic report. Sensitivity analyses excluding unverified outcomes and analyses of agreement with medical records may be required. Reassuringly, several studies have supported the ability of patients to accurately report their reproductive history, particularly with regards to surgical interventions, fertility treatments and pregnancy outcomes.^34 35^

### Power calculation and planned analysis

The target sample size was determined by a simulation-based power calculation. Data were simulated based on a mixed effects logistic regression model with a single fixed effect for treatment and a single random effect for participant. Different random effect SD of 0.5, 0.7 and 0.9 were considered, based on previous studies reporting random effects with SD around 0.7.^36^ Simulations used 5 cycles per participant for natural conception and 3 cycles per participant for IVF. For each set of simulation parameters, 1000 data sets were generated, mixed effects logistic regression models were fitted, and the power was calculated as the proportion of models where the p value for the main effect was below 0.05. The simulation was done using R V.4.0.2, with the glmmTMB package used to fit the mixed effects logistic regression models.

Natural conception based LBR of 1.4% per cycle has been reported in women with moderate to severe endometriosis and 12 months of unsuccessfully trying.^37^ An increase to at least 4% is needed to be clinically significant.^6^ A sample size of N=400 (200 per arm) would achieve over 90% power under the scenarios considered.

An IVF conception-based LBR of 12% per cycle has been reported in women with moderate to severe endometriosis.^38^ An increase to 20% would be needed to be clinically significant. A target sample size of N=300 (150 per arm) would achieve a power of 82% to 87% under the scenarios considered.

Demographic continuous variables will be compared using the Welch t-test. Proportions will be compared using χ^2^ test. P values below 0.05 will be considered statistically significant. The primary analysis will use discrete-time survival analysis to compare time to pregnancy and time to live birth between the two treatment groups, using inverse probability weighting to account for non-random dropout.^39^ As a sensitivity analysis, mixed effects logistic regression models will also be used to compare clinical pregnancy and LBRs between the two treatment groups, allowing for repeated measures (multiple cycles of attempted conception) within individual participants. Both approaches will adjust for expected confounders, including length of infertility, age, parity, gravidity, indicators of male-factor infertility on semen analysis and selected comorbidities. As this is a multisite study, between-site heterogeneity will be addressed analytically by incorporating site-level random effects. Missing data will be accounted for using multiple imputation by chained equations. The results from a complete case analysis will also be reported, as a sensitivity analysis.

A single, study-wide protocol has been implemented across all participating sites with uniform inclusion/exclusion criteria, standardised outcome definitions, specified data collection schedules (baseline, monthly cycle and operation questionnaires), imaging criteria for moderate/severe endometriosis, and centralised data capture via REDCap and the ELFS App to reduce risks of heterogeneity that could compromise internal validity and to minimise potential bias. Core datasets will be harmonised through project-specific REDCap baseline questionnaires and ELFS App cyclical questionnaires, with prespecified systematic reporting of imaging and surgical findings. All recruitment team members will receive centralised training on eligibility screening, participant interactions and ELFS App setup to ensure consistent participant engagement. Central monitoring will be conducted by the coordinating principal investigator and trials coordinator, supported by real-time recruitment and data-collection dashboards and calculators within the ELFS App.

### Patient and public involvement

None.

## Discussion

At present, it is unclear whether prepregnancy surgery for moderate or severe endometriosis, either in the setting of established infertility or even before a woman has begun trying to conceive, confers any benefit in terms of spontaneous or ART conception, live birth or other obstetric outcomes. The outcomes of this research aim to improve endometriosis management for women desiring pregnancy by establishing if there is a fertility benefit of prepregnancy surgery in patients with moderate or severe endometriosis.

Other authors are aiming to investigate the relationship between endometriosis, surgery, and subsequent fertility. To date, we are aware of three international randomised controlled trials currently recruiting. Ottolina *et al*^40^ plan to randomise infertile patients to either surgery (followed by attempts to spontaneously conceive) or IVF. They plan a pragmatic intention to treat analysis. The investigators hypothesise surgery will have a favourable outcome on pregnancy rate, but that IVF will be more successful, and have powered their study based on a 30% LBR in the surgery arm and 50% LBR in the IVF arm. In the Impact of Operation on Fertility for Women With Severe Endometriosis study,^41^ Raos *et al* plan to compare the impact of surgery and ART on fertility for women with deep infiltrating endometriosis of the rectosigmoid. The investigators will compare CPR and LBR 18 months after first-line surgery and first-line ART. In both these studies, investigators have anticipated highly favourable pregnancy rates in a population with either known infertility or high risk of subfertility,^15 17 42–44^ and have not considered the potential impact of comorbid adenomyosis in further reducing fecundity.^17 42 45 46^ The third randomised trial, the ENDOFERT Study, registered by Collinet *et al*^47^ (ClinicalTrials.gov reference number NCT02948972) continues to recruit. This study is randomising infertile participants to either surgery plus post-operative IVF or IVF alone, aiming to examine if pre-IVF surgery improves pregnancy rates.

While these trials will provide important randomised evidence on surgery versus ART in selected infertile or high-risk groups, their restrictive eligibility, omission of natural conception arms and lack of systematic assessment of comorbid adenomyosis limit generalisability. Moreover, these three study designs anticipate that participants will be willing to be randomised despite the scientific equipoise. Many patients with severe endometriosis, even in the absence of colorectal or urological obstruction (both of which are exclusion criteria in these randomised trials), will be highly symptomatic, and despite fertility desires, will actively pursue surgery in the hope of symptom control. These studies underscore the need to also measure natural time-to-pregnancy and spontaneous conception rates, and to adopt a broader, real-world design that captures symptom burden and patient treatment preferences alongside fertility outcomes.

Alongside these important ongoing randomised studies, ELFS aims to compare fertility outcomes for those who have moderate or severe endometriosis pursuing either natural or ART conception, including both those who undergo preconception surgery and those who are conservatively managed, with cross-comparisons between all four groups. The large sample size and pragmatic observational approach have, to date, demonstrated feasible recruitment.

This study has several important potential limitations. The primary outcome requires a large participant sample, but strict inclusion criteria to ensure accurate diagnosis of moderate and severe endometriosis may considerably restrict the eligible population. This could slow recruitment, reduce statistical power if target numbers are not met, and introduce selection bias, which in turn may limit applicability beyond specialist endometriosis care settings.

Furthermore, the repeated collection of sensitive data, including sexual history, fertility and pregnancy outcomes, may negatively affect participant experience and engagement. The burden and intrusiveness of such assessments could lead to distress, underreporting of sensitive information, and differential loss to follow-up, with potential for missing and biased data. Together, these factors may affect both the internal and external validity of the study’s findings.

### Dissemination of findings

Findings will be disseminated in a peer-reviewed journal and presented at national and international meetings. This project will deliver meaningful and important outcomes for Australian and international patients living with endometriosis and the clinicians who seek to provide best practice endometriosis care.

### Planned timetable and site(s) for the study

The official start date with first participant recruitment was 1 December 2021. The anticipated completion date is December 2027. Potential for additional international collaboration is in the planning stages. The study is sponsored by the Royal Women’s Hospital, Parkville Victoria. Current recruitment sites include The Royal Women’s Hospital, Mercy Hospital for Women, Western Health (Joan Kirner), Epworth Healthcare, Ramsay HealthCare, Melbourne IVF, NewLife IVF, City Fertility, Genea Fertility, Life Fertility, Albury Wodonga Health, Royal Prince Alfred Hospital and Hadassah Medical Center (Jerusalem). Approvals are pending to commence recruitment at Lyell McEwin Hospital, King Edward Memorial Hospital and Barwon Health in early 2026.

## References

[1] Bhattacharya S, Porter M, Amalraj E (2009). The epidemiology of infertility in the North East of Scotland. Hum Reprod.

[2] de Ziegler D, Borghese B, Chapron C (2010). Endometriosis and infertility: pathophysiology and management. *The Lancet*.

[3] Missmer SA, Hankinson SE, Spiegelman D (2004). Incidence of laparoscopically confirmed endometriosis by demographic, anthropometric, and lifestyle factors. Am J Epidemiol.

[4] Leone Roberti Maggiore U, Chiappa V, Ceccaroni M (2024). Epidemiology of infertility in women with endometriosis. Best Pract Res Clin Obstet Gynaecol.

[5] American Society for Reproductive Medicine (1997). Revised American Society for Reproductive Medicine classification of endometriosis: 1996. Fertil Steril.

[6] Marcoux S, Maheux R, Bérubé S (1997). Laparoscopic Surgery in Infertile Women with Minimal or Mild Endometriosis. *N Engl J Med*.

[7] Bafort C, Beebeejaun Y, Tomassetti C (2020). Laparoscopic surgery for endometriosis. Cochrane Database Syst Rev.

[8] Roman H, Chanavaz-Lacheray I, Ballester M (2018). High postoperative fertility rate following surgical management of colorectal endometriosis. Hum Reprod.

[9] Mooney SS, Ross V, Stern C (2021). Obstetric Outcome After Surgical Treatment of Endometriosis: A Review of the Literature. *Front Reprod Health*.

[10] Liang Y, Liu M, Zhang J (2024). First-line surgery versus first-line assisted reproductive technology for women with deep infiltrating endometriosis: a systematic review and meta-analysis. Front Endocrinol (Lausanne).

[11] Bourdon M, Peigné M, Maignien C (2024). Impact of Endometriosis Surgery on In Vitro Fertilization/Intracytoplasmic Sperm Injection Outcomes: a Systematic Review and Meta-analysis. Reprod Sci.

[12] Stepniewska A, Pomini P, Bruni F (2009). Laparoscopic treatment of bowel endometriosis in infertile women. Hum Reprod.

[13] Donnez J, Squifflet J (2010). Complications, pregnancy and recurrence in a prospective series of 500 patients operated on by the shaving technique for deep rectovaginal endometriotic nodules. Hum Reprod.

[14] Tuominen A, Saavalainen L, Tiitinen A (2021). Pregnancy and delivery outcomes in women with rectovaginal endometriosis treated either conservatively or operatively. Fertil Steril.

[15] Daniilidis A, Angioni S, Di Michele S (2022). Deep Endometriosis and Infertility: What Is the Impact of Surgery?. J Clin Med.

[16] Casals G, Carrera M, Domínguez JA (2021). Impact of Surgery for Deep Infiltrative Endometriosis before In Vitro Fertilization: A Systematic Review and Meta-analysis. J Minim Invasive Gynecol.

[17] Cohen J, Thomin A, Mathieu D’Argent E (2014). Fertility before and after surgery for deep infiltrating endometriosis with and without bowel involvement: a literature review. Minerva Ginecol.

[18] Becker CM, Bokor A, Heikinheimo O (2022). ESHRE guideline: endometriosis. Hum Reprod Open.

[19] Bafort C, Dancet E, Mellaerts J (2024). Correlation Between Surgical Phenotype and Pain Improvement After Endometriosis Surgery. J Minim Invasive Gynecol.

[20] Younis JS, Nelson SM What is the recommended management of a young woman with an intact endometrioma desiring future fertility?. Front Endocrinol.

[21] Coccia ME, Nardone L, Rizzello F (2022). Endometriosis and Infertility: A Long-Life Approach to Preserve Reproductive Integrity. Int J Environ Res Public Health.

[22] Borisova AV, Konnon SRD, Tosto V (2022). Obstetrical complications and outcome in patients with endometriosis. J Matern Fetal Neonatal Med.

[23] Lalani S, Choudhry AJ, Firth B (2018). Endometriosis and adverse maternal, fetal and neonatal outcomes, a systematic review and meta-analysis. Hum Reprod.

[24] Zullo F, Spagnolo E, Saccone G (2017). Endometriosis and obstetrics complications: a systematic review and meta-analysis. Fertil Steril.

[25] Alberico D, Somigliana E, Bracco B (2018). Potential benefits of pregnancy on endometriosis symptoms. Eur J Obstet Gynecol Reprod Biol.

[26] Fernando S, Breheny S, Jaques AM (2009). Preterm birth, ovarian endometriomata, and assisted reproduction technologies. Fertil Steril.

[27] Shahmoradi F, Haghighi L, Noori M (2024). Endometriosis and adverse pregnancy outcomes: A case-control study. *Int J Reprod Biomed*.

[28] Stephansson O, Kieler H, Granath F (2009). Endometriosis, assisted reproduction technology, and risk of adverse pregnancy outcome. Hum Reprod.

[29] Harris PA, Taylor R, Minor BL (2019). The REDCap consortium: Building an international community of software platform partners. J Biomed Inform.

[30] Harris PA, Taylor R, Thielke R (2009). Research electronic data capture (REDCap)--a metadata-driven methodology and workflow process for providing translational research informatics support. J Biomed Inform.

[31] Hudelist G, Montanari E, Salama M (2021). Comparison between Sonography-based and Surgical Extent of Deep Endometriosis Using the Enzian Classification - A Prospective Diagnostic Accuracy Study. J Minim Invasive Gynecol.

[32] Hjermstad MJ, Fayers PM, Haugen DF (2011). Studies comparing Numerical Rating Scales, Verbal Rating Scales, and Visual Analogue Scales for assessment of pain intensity in adults: a systematic literature review. J Pain Symptom Manage.

[33] Proctor M, Farquhar C (2006). Diagnosis and management of dysmenorrhoea. *BMJ*.

[34] Shafrir AL, Wise LA, Palmer JR (2021). Validity of self-reported endometriosis: a comparison across four cohorts. Human Reproduction.

[35] Stern JE, McLain AC, Buck Louis GM (2016). Accuracy of self-reported survey data on assisted reproductive technology treatment parameters and reproductive history. Am J Obstet Gynecol.

[36] Ecochard R, Clayton DG (1998). Multi-level modelling of conception in artificial insemination by donor. Stat Med.

[37] Leone Roberti Maggiore U, Scala C, Tafi E (2017). Spontaneous fertility after expectant or surgical management of rectovaginal endometriosis in women with or without ovarian endometrioma: a retrospective analysis. Fertil Steril.

[38] Ashrafi M, Arabipoor A, Hemat M (2019). The impact of the localisation of endometriosis lesions on ovarian reserve and assisted reproduction techniques outcomes. J Obstet Gynaecol.

[39] Modest AM, Wise LA, Fox MP (2018). IVF success corrected for drop-out: use of inverse probability weighting. Hum Reprod.

[40] Ottolina J, Vignali M, Papaleo E (2022). Surgery versus IVF for the treatment of infertility associated to ovarian and deep endometriosis (SVIDOE: Surgery Versus IVF for Deep and Ovarian Endometriosis). Clinical protocol for a multicenter randomized controlled trial. PLoS ONE.

[41] Raos M, Roman H, Seyer-Hansen M (2022). EFFORT study: Comparing impact of operation and assisted reproductive technologies on fertility for women with deep infiltrating endometriosis - study protocol for a multicentre randomised trial. BMJ Open.

[42] Iversen ML, Seyer-Hansen M, Forman A (2017). Does surgery for deep infiltrating bowel endometriosis improve fertility? A systematic review. Acta Obstet Gynecol Scand.

[43] Vercellini P, Somigliana E, Viganò P (2009). Surgery for endometriosis-associated infertility: a pragmatic approach. Hum Reprod.

[44] Raos M, Mathiasen M, Seyer-Hansen M (2023). Impact of surgery on fertility among patients with deep infiltrating endometriosis. Eur J Obstet Gynecol Reprod Biol.

[45] Tomassetti C, Meuleman C, Timmerman D (2013). Adenomyosis and subfertility: evidence of association and causation. Semin Reprod Med.

[46] Daraï E, Cohen J, Ballester M (2017). Colorectal endometriosis and fertility. Eur J Obstet Gynecol Reprod Biol.

[47] Collinet P (2026). Impact of complete surgery of colorectal deep infiltrating endometriosis on fertility (ENDOFERT). clinicaltrials.gov identifier: NCT02948972. NCT02948972.

