# Supplementary material for: Endometriosis Longitudinal Fertility Study (ELFS): a prospective longitudinal cohort study of the effect of conservative and surgical management for moderate to severe endometriosis on future fertility – study protocol
Source: BMJ Open. 2026 Aug 20;16(8):e114124. doi: 10.1136/bmjopen-2025-114124 (PMC13504474; doi:10.1136/bmjopen-2025-114124)
Supplement: Supplementary file 1 [file bmjopen-2025-114124.reviewer_comments.pdf]

PEER REVIEW HISTORY

BMJ Open publishes all reviews undertaken for accepted manuscripts. Reviewers are asked to complete a checklist review form and are provided with free text boxes to elaborate on their assessment. These free text comments are reproduced below.

ARTICLE DETAILS

Title (Provisional)

The Endometriosis Longitudinal Fertility Study (ELFS): A prospective longitudinal cohort study of the effect of conservative and surgical management for moderate to severe endometriosis on future fertility -Study Protocol

Authors

Mooney, Samantha; Ross, Vanessa; Dior, Uri P.; Reddington, Charlotte; Hicks, Lauren; Cheng, Claudia; Amir, Michal; Harlow, Keryn; Holdsworth-Carson, Sarah; Stern, Kate; Healey, Martin; Rogers, Peter; END-IT Endometriosis Research Group, END-IT Endometriosis Research Group

---

VERSION 1 - REVIEW

Reviewer 1

Name Gajbhiye, Rahul

Affiliation ICMR - National Institute for Research in Reproductive and Child Health, Department of Clinical Research

Date 19-Feb-2026

COI None

---

1. The Introduction needs to be more focused and directly aligned with the study objectives. At present, it reads like a narrative review with excessive background detail. The authors should remove non-essential information and retain only the evidence that clearly supports the rationale for their research question. The section should more clearly identify the knowledge gap and explain how the present study addresses it.

2. Page 7, line 9, “The statement that infertility is associated with an increased risk of endometriosis diagnosis” requires clarification.

Endometriosis is itself a known cause of infertility, and hence directionality is complex. Women undergoing diagnostic laparoscopy for infertility evaluation may increase detection rates (ascertainment bias). The authors should clarify whether they are referring to biological risk, detection probability, or odds of diagnosis, and discuss the potential for reverse causation and diagnostic bias.

3. Line 7-11. Kindly specify which outcome was improved (clinical pregnancy rate or live birth rate).

4. Line 16-32. The paragraph is overly detailed and reads like a summary of multiple papers rather than a focused synthesis. It should be condensed. Authors should highlight only the key findings relevant to their research question, rather than describing individual study characteristics in detail.

5. Evidence on pregnancy outcomes in endometriosis has also been reported from low- and middle-income countries (LMICs). The authors should include relevant references from these settings to provide a more balanced and globally representative perspective. This will strengthen the background section and improve the study's contextual relevance.

6. Page 8, lines 16-18, The study groups are not clearly described. Please revise the sentence to clearly define the four comparison groups and avoid ambiguous phrasing such as "in situ … pursuing conception."

7. Objectives need to be clearly and specifically defined. Please distinguish primary and secondary outcomes and clearly describe the comparison groups.

8. Methods: From the description, this appears to be a multinational and multi-centre study involving both public and private institutions across Australia and Israel. The manuscript does not clearly explain how uniform data collection will be ensured across different centres and countries. Given that clinical management protocols, ART practices, surgical approaches, and documentation standards may vary between institutions and countries, the authors should clarify:

a. Whether a standardized study protocol will be used across all sites

b. How data collection tools will be harmonized

c. Whether investigators will receive centralized training

d. How differences in clinical management will be accounted for in analysis

e. Whether there will be central monitoring or data auditing

Without this clarification, there is a risk of heterogeneity affecting internal validity.

9. There is inconsistent use of terminology. In some places, the manuscript refers to "patients," while elsewhere it uses "participants." The authors should use consistent terminology throughout, preferably "participants" once individuals are enrolled in the study.

10. The study participants recruitment process is broad but insufficiently controlled. The authors need to clarify standardization, consent safeguards, and potential bias control.

11. The role of male factor infertility has not been addressed in the inclusion/exclusion criteria. Since pregnancy outcomes depend on both partners, failure to account for male factor infertility may significantly confound the results.

12. The rASRM staging system is a surgical classification and cannot be accurately assigned based on ultrasound or MRI findings alone. The authors should clarify how stage III–IV disease will be classified in participants without prior laparoscopy. If imaging-based criteria are being used, an appropriate imaging classification system (e.g., Enzian classification) should be adopted instead of rASRM staging.

13. The protocol does not clearly describe how the data collection tools were developed or validated. Details regarding training of research staff, standardization procedures, and quality control measures are missing. Since participants are expected to enter their own clinical data, the authors must clarify how data accuracy will be verified and how discrepancies will be managed. A clear data management and monitoring plan is required to ensure reliability and reduce bias.

14. The Discussion section requires refinement. It should be more focused and closely aligned with the stated study objectives and expected outcomes. Currently, it appears broad and somewhat narrative.

VERSION 1 - AUTHOR RESPONSE

Reviewer: 1
Dr. Rahul Gajbhiye, ICMR - National Institute for Research in Reproductive and Child Health
Comments to the Author:

1. The Introduction needs to be more focused and directly aligned with the study objectives. At present, it reads like a narrative review with excessive background detail. The authors should remove non-essential information and retain only the evidence that clearly supports the rationale for their research question. The section should more clearly identify the knowledge gap and explain how the present study addresses it.

We thank you for this comment. We have condensed the introduction and retained what we believe to be relevant background to support the aims of our study. See pages 5-6, lines 66-123.

2. Page 7, line 9, "The statement that infertility is associated with an increased risk of endometriosis diagnosis" requires clarification.
Endometriosis is itself a known cause of infertility, and hence directionality is complex. Women undergoing diagnostic laparoscopy for infertility evaluation may increase detection rates (ascertainment bias). The authors should clarify whether they are referring to biological risk, detection probability, or odds of diagnosis, and discuss the potential for reverse causation and diagnostic bias.

Thank you — we agree that the directionality between endometriosis and infertility is complex and that diagnostic ascertainment can bias observed associations. Our intent was to report the association between infertility and subsequent endometriosis diagnosis in the epidemiologic literature, not to imply a single causal direction. We have

clarified this in the opening paragraph. We believe, however, discussion to explicitly address causal links and even reverse causation, diagnostic (ascertainment) bias from infertility workups (including laparoscopy), and the distinction between biological risk versus increased detection/odds of diagnosis may be beyond the scope of this protocol paper. See page 5, lines 66-69.

3. Line 7-11. Kindly specify which outcome was improved (clinical pregnancy rate or live birth rate).

Thank you for requesting clarification. The study by Marcoux et al reports improved spontaneous pregnancy rates in infertile women with minimal or mild endometriosis who undergo surgical treatment prior to attempted conception. The Cochrane review by Bafort et al reports increased clinical pregnancy rate and ongoing pregnancy rates for women with minimal–mild endometriosis who undergo surgery. We have made this clearer in the manuscript. See page 5, line 71.

4. Line 16-32. The paragraph is overly detailed and reads like a summary of multiple papers rather than a focused synthesis. It should be condensed. Authors should highlight only the key findings relevant to their research question, rather than describing individual study characteristics in detail.

Thank you for the comment. We have condensed this as described above.

5. Evidence on pregnancy outcomes in endometriosis has also been reported from low- and middle-income countries (LMICs). The authors should include relevant references from these settings to provide a more balanced and globally representative perspective. This will strengthen the background section and improve the study's contextual relevance.

Thank you for this comment. If the referenced systematic reviews, several included studies were performed in middle-income countries (predominantly China and Taiwan). I've included a more recent study from Iran (Shahmoradi et al, 2024), currently classed as a low-middle income country by the World Bank. We are not aware of any studies conducted in low income countries examining the relationship between endometriosis and pregnancy outcomes. See page 5, line 105.

6. Page 8, lines 16-18, The study groups are not clearly described. Please revise the sentence to clearly define the four comparison groups and avoid ambiguous phrasing such as "in situ ... pursuing conception."

Thank you for this comment. We have reworded the description of the study groups. See page 6, lines 112-117. We have also more specifically described the cohorts in the methods under sample selection. See pages 8-9, lines 225-231.

7. Objectives need to be clearly and specifically defined. Please distinguish primary and secondary outcomes and clearly describe the comparison groups.

We have added more clarity and aligned these with our clinical trials registration. See pages 6-7, line 137-164.

8. Methods: From the description, this appears to be a multinational and multi-centre study involving both public and private institutions across Australia and Israel. The manuscript does not clearly explain how uniform data collection will be ensured across different centres and countries. Given that clinical management protocols, ART practices, surgical approaches, and documentation standards may vary between institutions and countries, the authors should clarify:
a. Whether a standardized study protocol will be used across all sites
b. How data collection tools will be harmonized
c. Whether investigators will receive centralized training.
d. How differences in clinical management will be accounted for in analysis
e. Whether there will be central monitoring or data auditing
Without this clarification, there is a risk of heterogeneity affecting internal validity.

a. Whether a standardized study protocol will be used across all sites

- Yes. A single, study-wide protocol will be used at all participating sites with defined inclusion/exclusion criteria, standardized outcome definitions, specified data collection schedules (baseline, monthly cycle questionnaires, operation questionnaire), imaging criteria for evidence of moderate/severe endometriosis, and use of a central REDCap database and ELFS App for data capture.

b. How data collection tools will be harmonized
Core datasets will be collected via project-specific baseline questionnaire via REDCap and cyclical questionnaires via the ELFS App. This ensures harmonized questions, formats, and definitions across sites. Imaging and surgical findings are requested in a prespecified, systematic way.

c. Whether investigators will receive centralized training.
The research team members involved in recruitment all receive centralised training with regarding to eligibility screening, participant interactions and ELFS App set-up and engagement. This is aimed to ensure standardised engagement for all participants.

d. How differences in clinical management will be accounted for in analysis
We anticipate there may be differences between Australian sites, and Australian and Israeli sites. We have tried to minimise heterogeneity by being strict with regards to the surgical expertise and ART availability across our study sites. However, we recognise we may need to account for site differences in our analysis. This will involve assessing effect heterogeneity, quantifying between-site variance and performing sensitivity analyses. If indicated, we plan to account for site by including site indicators (fixed effects) or random-effects/multilevel models for estimates, and incorporate site in propensity-scoring if necessary.

e. Whether there will be central monitoring or data auditing

Data will be monitored centrally by the coordinating principal investigator and trials coordinator. The ELFS App has inbuilt calculators available on a research team dashboard to allow real-time updates in recruitment, and cyclic data collection.

We have added a paragraph summarising this (page 11, lines 322-335):
A single, study-wide protocol will be implemented across all participating sites with uniform inclusion/exclusion criteria, standardized outcome definitions, specified data collection schedules (baseline, monthly cycle and operation questionnaires), imaging criteria for moderate/severe endometriosis, and centralized data capture via REDCap and the ELFS App to reduce risks of heterogeneity that could compromise internal validity. Core datasets will be harmonized through project-specific REDCap baseline questionnaires and ELFS App cyclical questionnaires, with prespecified systematic reporting of imaging and surgical findings. All recruitment team members will receive centralized training on eligibility screening, participant interactions, and ELFS App setup to ensure consistent participant engagement. Anticipated between-site differences (Australian vs Israeli and across Australian centres) will be addressed analytically by assessing effect heterogeneity, quantifying between-site variance, performing sensitivity analyses, and—if needed—incorporating site indicators or multilevel/random-effects models and site-informed propensity scoring. Central monitoring will be conducted by the coordinating principal investigator and trials coordinator, supported by real-time recruitment and data-collection dashboards and calculators within the ELFS App.

9. There is inconsistent use of terminology. In some places, the manuscript refers to "patients," while elsewhere it uses "participants." The authors should use consistent terminology throughout, preferably "participants" once individuals are enrolled in the study.

We have removed reference to "patients" in the methods, and replaced with participants. The word "patient" remains in other sections as is appropriate.

10. The study participants recruitment process is broad but insufficiently controlled. The authors need to clarify standardization, consent safeguards, and potential bias control.

Thank you for this important comment. We have implemented standardized enrolment and data procedures including a written inclusion/exclusion checklist used by all screeners, a centralized electronic case report form with a data dictionary, version control and automated validation, and a uniform baseline template capturing demographics, menstrual and reproductive history, medications, and comorbidities. Primary and secondary endpoints (clinical pregnancy, live birth etc) were prespecified with explicit operational definitions. Consent procedures emphasized autonomy and privacy by separating consent discussions from clinical visits and obtaining separate consent for partner data/samples. To mitigate bias we are recruit across diverse settings to reduce referral bias, published transparent eligibility criteria and screening logs, collected key confounders (age, BMI, smoking, prior treatments, ovarian reserve

markers) for prespecified adjustment, and defined a missing-data plan (sensitivity analyses).

We have commented on these points in the manuscript.

11. The role of male factor infertility has not been addressed in the inclusion/exclusion criteria. Since pregnancy outcomes depend on both partners, failure to account for male factor infertility may significantly confound the results.

We thank the reviewer for this comment. We are collecting data on male-factor infertility (in the form of semen analysis results and possible donor use. We have added mention of this in the manuscript. Pages 7-8, lines 186-188.

12. The rASRM staging system is a surgical classification and cannot be accurately assigned based on ultrasound or MRI findings alone. The authors should clarify how stage III–IV disease will be classified in participants without prior laparoscopy. If imaging-based criteria are being used, an appropriate imaging classification system (e.g., Enzian classification) should be adopted instead of rASRM staging.

We thank the reviewer for this important point. The main issue is, in 2019-2020 when this study protocol was developed, there was no non-surgical "pre-operative" grading system validated in the literature. The sonographic #Enzian classification system was published and validated in 2021 (Keckstein et al., 2021), just as recruitment for ELFS began. In Australia and Israel, the rASRM classification is used intraoperatively, with the EFI applied postoperatively to patients trying to conceive. A pragmatic decision was made to use imaging (expert pelvic ultrasound or MRI) to define our study population with evidence of moderate or severe endometriosis. Using surgical diagnosis only would have significant ramifications for recruitment, as it is uncommon in our clinical practice to perform diagnostic laparoscopy alone. We recognise the use of the rASRM criteria to imaging is not validated, and thus is a limitation of our study design. However, due to strict protocols regarding eligible imaging providers, and findings that meet the criteria for moderate or severe endometriosis, we are confident we are recruiting an appropriate cohort. We plan to analyse the surgical cohort to confirm recruitment of patients who meet rASRM grade 3 and 4. Given the same imaging criteria apply to all enrolled participants, this will support our assertion that the non-surgical group also meet the definitions for rASRM grade 3 and 4 (as supported by the study by Hudelist et al, 2021).

We have inserted a brief paragraph on this. See page 8, lines 215-223.

13. The protocol does not clearly describe how the data collection tools were developed or validated. Details regarding training of research staff, standardization procedures, and quality control measures are missing. Since participants are expected to enter their own clinical data, the authors must clarify how data accuracy will be verified and how discrepancies will be managed. A clear data management and monitoring plan is required to ensure reliability and reduce bias.

Details describing data collection tools, research team training and standardisation of procedures has now been provided as previously mentioned.

We agree that clarity about how participant-entered clinical data will be verified is essential. We use multiple complementary measures to ensure accuracy of participant-entered clinical data: structured questionnaire items and real-time front-end checks to prevent errors; automated rules that flag implausible or inconsistent responses with in-app prompts for confirmation; staff follow-up (email/phone/in-app) and optional participant uploads of corroborating documents; medical-record verification for consenting participants to quantify agreement and inform sensitivity analyses; a predefined workflow for discrepancy resolution including staff review and clinician adjudication; and full audit trails of all entries/edits. These steps, combined with pilot-tested instructions and staff training, minimize errors and allow transparent handling and analysis of any remaining discrepancies. We have added a succinct paragraph to describe this. See page 10, lines 288-293.

14. The Discussion section requires refinement. It should be more focused and closely aligned with the stated study objectives and expected outcomes. Currently, it appears broad and somewhat narrative.

We have aimed to narrow this discussion and focus on the ELFS objectives.

VERSION 2 - REVIEW

Reviewer 1

Name Gajbhiye, Rahul

Affiliation ICMR - National Institute for Research in Reproductive and Child Health, Department of Clinical Research

Date 22-Apr-2026

COI

The authors have made sincere efforts to address reviewer comments, particularly by improving clarity around imaging-based classification, data collection procedures, and multicentre standardisation. These additions enhance the transparency of the protocol.

Specific comments:

• The observational comparison between surgical and conservative management is highly susceptible to confounding by indication, and the current analytical approach does not adequately address this. Similarly, the use of per-cycle outcomes without a clearly defined time-to-event framework raises concerns regarding bias and interpretability. In addition, a detailed strategy for handling missing data in this longitudinal design is lacking.

• Although data collection on Male factor infertility is mentioned (response to comments), there is no clear indication of how this will be incorporated into the analysis. This needs to be explicitly included in the analytical framework.

• Additional details on Data validation and app-based collection have been provided, which is an improvement. However, clarity is still needed on:

1. extent of medical record verification

2. proportion of validated outcomes

• The added description on Multicentre standardisation is useful, but still largely descriptive. More clarity is required on how inter-site variability will be quantitatively handled.

• There are still typographical and formatting issues in the manuscript (e.g., text inconsistencies and sentence breaks in multiple sections, including Strengths & Limitations, page 4). This needs careful proofreading.

Overall, while the manuscript has improved in clarity, methodological limitations remain, which require further strengthening before the protocol can be considered robust.

VERSION 2 - AUTHOR RESPONSE

Comments to Author: Author Responses

1. The observational comparison between surgical and conservative management is highly susceptible to confounding by indication, and the current analytical approach does not adequately address this. Similarly, the use of per-cycle outcomes without a clearly defined time-to-event framework raises concerns regarding bias and interpretability. In addition, a detailed strategy for handling missing data in this longitudinal design is lacking.

We have clarified the inclusion of confounders as covariates in the regression model, and acknowledge that residual confounding is always a concern with an observational study. We note that the choice of per-cycle logistic regression or time-to-event models in this context has been discussed at length in the literature, with both choices having advantages and disadvantages. The original choice of mixed effects logistic regression was made to avoid concerns about standard time-to-event models assuming that drop-out (censoring) is completely at random, an assumption which we believe is untenable in this context. Thanks to your review raising this matter again, we have found alternative time-to-event models that account for informative censoring (Modest et al 2018) and have adjusted our analysis plan accordingly.

See page 11, lines 332-343.
"Demographic continuous variables will be compared using the Welch t-test. Proportions will be compared using Chi-square test. P-values below 0.05 will be considered statistically significant. The primary analysis will use discrete-time survival analysis to compare time to pregnancy and time to live birth between the two treatment groups, using inverse probability weighting to account for non-random dropout.[39] As a sensitivity analysis, mixed effects logistic regression models will also be used to compare clinical pregnancy and live birth rates between the 2 treatment groups, allowing for repeated measures (multiple cycles of attempted conception) within individual participants. Both approaches will adjust for expected confounders, including length of infertility, age, parity, gravidity, indicators of male-factor infertility on semen analysis and selected comorbidities. As this is a multi-

site study, between-site heterogeneity will be addressed analytically by incorporating site-level random effects. Missing data will be accounted for using multiple imputation by chained equations ("MICE"). The results from a complete case analysis will also be reported, as a sensitivity analysis."

2. Although data collection on Male factor infertility is mentioned (response to comments), there is no clear indication of how this will be incorporated into the analysis. This needs to be explicitly included in the analytical framework.
The use of male factor infertility has now been addressed in the statistical methods section.
See page 11, line 339.

3. Additional details on Data validation and app-based collection have been provided, which is an improvement. However, clarity is still needed on:
a) extent of medical record verification. Thank you for the comment. We have added brief additional clarity on page 10, lines 298-306. We believe that the App provides an extremely valuable tool for patient reported outcome measures, including pain symptoms, menstrual cycle experiences, and cycle-to-cycle fertility outcomes.
"For those undergoing surgery during the study period, surgical reports will be collected and assessed by one of two investigators (SM and VR); any disagreements will be clarified by investigator MH. The primary outcome, live birth rate, is assessed by patient reported data (via the ELFS App) and cross-validated against obstetric outcome data retrieved from the hospital in which the birth occurs. Similarly, clinical pregnancy rate is assessed by patient report, and validated by review of first trimester sonographic report. Sensitivity analyses excluding unverified outcomes and analyses of agreement with medical records may be required. Reassuringly, several studies have supported the ability of patients to accurately report their reproductive history, particularly with regards to surgical interventions, fertility treatments and pregnancy outcomes.[34, 35]"

b) proportion of validated outcomes. We value this query. Live birth rate (LBR) is the primary outcome and is defined in the methods. This is a valid and important outcome in fertility research. We are collecting primary outcome data from patient report, and then cross-validating this with obstetric outcome data directly from the hospital records where the birth occurs. Secondary outcomes include clinical pregnancy rate (CPR), which is also defined in the methods. We are also collecting pain scores by using a 4-point verbal rating score (VRS), chosen for its simplicity, particularly as participants are asked about their pain experience every cycle/month. These secondary outcomes are collected by patient report. The research team also validate the patient report of CPR by collecting sonographic reports. We have clarified this in the methods. As described above, these changes are made on page 10, lines 298-306.

4. The added description on Multicentre standardisation is useful, but still largely descriptive. More clarity is required on how inter-site variability will be quantitatively handled.
The statistical methods sections has been updated to specify how heterogeneity between sites will be accounted for in the analysis. We hope this provides sufficient clarity and precision.
See page 11, lines 339-342.

5. There are still typographical and formatting issues in the manuscript (e.g., text inconsistencies and sentence breaks in multiple sections, including Strengths & Limitations, page 4). This needs careful proofreading.
Thank you for your comment. We've carefully re-proof-read the work. We are concerned there may have been a formatting/transition problem with the upload software, as on our version of Word the sentence breaks are not problematic/consistent. We will upload a clean pdf document as well, in case the issue happens again.
